# Low fasting serum insulin and dementia in nondiabetic women followed for 34 years

**DOI:** 10.1212/WNL.0000000000005911

**Published:** 2018-07-31

**Authors:** Kirsten Mehlig, Leif Lapidus, Dag S. Thelle, Margda Waern, Henrik Zetterberg, Cecilia Björkelund, Ingmar Skoog, Lauren Lissner

**Affiliations:** From the Institutes of Medicine (K.M., L.L., D.S.T., C.B., L.L.) and Neuroscience and Physiology (M.W., H.Z., I.S.), University of Gothenburg, Sweden; Institute of Basic Medical Sciences (D.S.T.), University of Oslo, Norway; UCL Institute of Neurology (H.Z.), Queen Square; UK Dementia Research Institute (H.Z.), London; and Clinical Neurochemistry Laboratory (H.Z.), Sahlgrenska University Hospital, Mölndal, Sweden.

## Abstract

**Objective:**

In a representative population of women followed over 34 years, we investigated the prospective association between fasting serum insulin and dementia, taking into account the incidence of diabetes mellitus.

**Methods:**

Fasting values for serum insulin and blood glucose were obtained in 1,212 nondiabetic women 38 to 60 years of age at the 1968 baseline. Risk of dementia was assessed by Cox proportional hazard regression with adjustment for insulin, glucose, and other covariates and, in a second model, after censoring for incident cases of diabetes mellitus. Incident diabetes mellitus was considered as a third endpoint for comparison with dementia.

**Results:**

Over 34 years, we observed 142 incident cases of dementia. The low tertile of insulin displayed excess risk for dementia (hazard ratio [HR] 2.34, 95% confidence interval [CI] 1.52–3.58) compared to the medium tertile, but the high tertile of insulin did not (HR 1.28, 95% CI 0.81–2.03). These associations were also seen for dementia without diabetes comorbidity. In contrast, high but not low insulin predicted incident diabetes mellitus (115 cases) (HR 1.70, 95% CI 1.08–2.68 and HR 0.76, 95% CI 0.43–1.37, respectively).

**Conclusion:**

A previous study reported a U-shaped association between fasting insulin and dementia in a 5-year follow-up of elderly men. Our results confirmed a nonlinear association in a female population, with high risk at low insulin values that was not attributable to preclinical dementia or impaired insulin secretion. This condition suggests a new pathway to dementia, which differs from the metabolic pathway involving diabetes mellitus.

Epidemiologic studies have observed associations between fasting insulin levels and risk of dementia, almost exclusively focusing on the high end of the insulin distribution and considering mainly the role of type 2 diabetes mellitus (T2DM).^1–4^ However, one study of elderly community-dwelling men followed up over 5 years showed that fasting insulin levels were related to risk of dementia in a U-shaped functional form, with elevated hazard ratios (HRs) at both ends of the insulin spectrum.^5^ Sensitivity analyses examined reverse causation, i.e., preclinical dementia causing low insulin values, but the association with low insulin levels remained. Produced in the pancreatic β cells, insulin enters the brain by a receptor-mediated, saturable transport mechanism, and brain insulin levels are proportional to peripheral levels under physiologic conditions.^6–9^ A U-shaped association between serum insulin and dementia is therefore plausible on the basis of clinical evidence regarding hyperinsulinemia^10^ and insulin deficiency in the brain.^7,11,12^ It has been pointed out that the latter could also be a consequence of previous peripheral hyperinsulinemia and T2DM.^7,8,13^

The purpose of this study was to investigate the association between fasting insulin and dementia in a female population followed over 34 years. The long observation period reduces the risk of reverse causation, i.e. the levels of fasting insulin being influenced by preclinical disease. Importantly, we take into account information on prevalent and incident diabetes diagnoses that were previously shown to predict dementia in these women.^14^ Using the same cohort and follow-up information, we aim to investigate whether both high and low fasting serum insulin levels in midlife predict dementia and how this association is related to diabetes comorbidity.

## Methods

### Study population

In 1968, a representative sample of 1,622 women 38, 46, 50, 54, or 60 years of age and living in Gothenburg, Sweden, were invited to the Prospective Population Study of Women in Gothenburg.^15^ A total of 1,462 women (90%) accepted the invitation and attended comprehensive physical and psychiatric examinations, at which they completed questionnaires about education, lifestyle, and medical history. Follow-up examinations were carried out in 1974, 1980, 1992, and 2000, with participation rates among those still alive decreasing from 91% in 1968 to 71% in 2000.^16,17^

### Standard protocol approvals, registrations, and patient consents

Participants provided informed oral consent in 1968, 1974, and 1980 and written consent in 1992 and later. Since 1980, all examinations have been approved by the Regional Ethics Review Board in Gothenburg in accordance with the Declaration of Helsinki.

### Endpoint definitions

The diagnosis of dementia was based on the combined information from psychiatric examinations and close informant interviews according to the DSM-III-R and on diagnostic data from the hospital discharge register and medical records from inpatient and outpatient departments and general practitioners' offices in Gothenburg for all women, including those lost to follow-up.^18^ Dementia subtypes were determined by geriatric psychiatrists and categorized as probable or possible Alzheimer disease (AD), vascular dementia (VD), or other dementia.^19^ Dementia diagnoses until December 31, 2002, were included in this report.

Diabetes mellitus was assessed at baseline and at each follow-up examination, and its incidence was recorded until the end of 2002.^20^ A woman was defined as having diabetes mellitus if the diagnosis was made by a physician, if she was on antidiabetic medication, or if her fasting blood glucose concentration was ≥7.0 mmol/L, in accordance with the current World Health Organization definition of diabetes mellitus.^21^ The information from examinations was checked for consistency with information from the Swedish Patient Register. Survivors who did not attend a certain follow-up examination were asked to complete a short postal questionnaire that included a question concerning diabetes status. Except for 2 cases of type 1 diabetes mellitus at baseline, all incident cases were T2DM.

### Baseline variables

At baseline (1968), venous blood samples were taken in all participants after an overnight fast, and fasting glucose was determined with a ferric cyanide reduction method adapted for autoanalyzer by Technicon.^22^ Fasting insulin was measured in serum samples from 1968 stored at −20°C for 45 years and validated by comparison with values that had been analyzed at baseline in a subsample of participants using a double antibody method.^23^ The analysis was performed in the Clinical Chemistry Laboratory at Sahlgrenska University Hospital, Mölndal, Sweden, with Elecsys kits on a Cobas 6000 analyzer (Roche Diagnostics, Basel, Switzerland) by board-certified laboratory technicians blinded to clinical data. Because absolute values were generally low, indicating an underestimation of the original values, we performed a nonparametric analysis based on tertiles that focused on the nonlinear relationship with risk of dementia. Secondary analyses were based on calibrated insulin values that were obtained from comparison of mean values in frozen relative to fresh samples, yielding a calibration factor of 1.88.^23^ These calibrated insulin values were used to derive approximate cut points characterizing the nonlinear association with risk of dementia and to estimate insulin sensitivity and β-cell function according to the homeostatic model assessment (HOMA) based on the updated computer model.^24,25^

Body mass index (BMI) was calculated from weight and height measured by standardized protocols.^15^ Obesity was defined as BMI ≥30 kg/m^2^. Hypertension was defined as systolic or diastolic blood pressure ≥140 or 90 mm Hg, respectively, in the sitting position after 5 minutes of rest or use of antihypertensive treatment. Total cholesterol, triglycerides, and leptin were measured in baseline blood samples as described previously.^23,26^ Hypertriglyceridemia was defined as triglyceride values ≥1.7 mmol/L.^27^ Higher education was defined as more than basic education. Parental history of diabetes mellitus was based on self-reported information collected at all examinations (at least 1 parent with diabetes vs none). Self-rated leisure time physical activity (LTPA) was assessed in 3 categories distinguishing low (sedentary lifestyle), moderate, and high LTPA.^28^ Smoking was assessed as current or previous smoking during the last 10 years vs never smoking or giving up smoking >10 years ago. Consumption of beer, wine, and spirits was dichotomized into current vs never or former use of each beverage. In 502 women participating in the fourth follow-up examination in 2000,^29^ *APOE* genotype was determined and parametrized in terms of the number of ε4 alleles per participant.

### Analytic sample

Prevalent cases of diabetes mellitus at baseline (n = 14) were excluded from the analysis because treatment may have affected the levels of insulin and glucose. Because no more serum samples from 1968 were available for 230 participants, the analytic sample was based on 1,218 women free of diabetes mellitus with values for insulin and glucose, which was reduced to 1,212 observations as a result of missing values for education or alcohol consumption. Among these, 142 women developed dementia before 2003, with a mean follow-up time to diagnosis of 25 years (SD = 6.1 years). The subtypes AD (n = 92) and VD (n = 66) were not mutually exclusive; 31 women had both diagnoses. The remaining 15 cases were other subtypes of dementia.^19^ Nineteen of 142 cases of dementia also had diabetes mellitus, and 4 of these were diagnosed within 9 years after dementia diagnosis. To separately investigate dementia diagnoses that were not mediated by diabetes mellitus, we considered dementia without any diagnosis of diabetes mellitus as an additional endpoint (n = 123), treating dementia with diabetes comorbidity as a competing event. The competing events included diabetes mellitus after the dementia diagnosis because the mean time difference of 3.8 years (range 1–9 years) was short compared to the latency period of dementia and because diabetes mellitus may be underdiagnosed in individuals with dementia. Because HOMA insulin sensitivity and β-cell function were defined only for glucose in (3.0–25.0) mmol/L and insulin in (2.88–57.6) mIU/L, the number of observations was reduced to 1,169 in regression models including these variables.

### Statistical methods

Fasting insulin was parameterized in terms of tertiles, which allowed us to examine the nonlinear association with dementia with consideration of the limited number of cases. Because of its correlation with insulin (*r*^2^ = 0.30, *p* < 0.001), fasting glucose was included as a potential confounder and parameterized in terms of tertiles as well. Cut points based on the analytic sample were given by 10.3 and 14.8 mIU/L (insulin) and 3.8 and 4.3 mmol/L (glucose). Risk of dementia was assessed by Cox proportional hazard regression mutually adjusted for tertiles of insulin and glucose, and for important covariates. Because age is the strongest risk factor for dementia, age at diagnosis or censoring was chosen as the relevant time scale, with left truncation at age at the baseline examination. To examine the risk for dementia without diabetes comorbidity, observations with both dementia and diabetes diagnosis were censored at the age at the earlier diagnosis. To examine the association with dementia subtypes, proportional hazard regression on tertiles of insulin and glucose was performed with stepwise forward selection of covariates (*p* for inclusion = 0.1, *p* for staying in the model = 0.05). Proportional hazard regression was also applied to investigate the effect of insulin and glucose on the risk for diabetes mellitus. Covariates included education, BMI, BMI squared, categories of LTPA, alcohol consumption, smoking status, and heredity for diabetes mellitus (for diabetes endpoints only). In sensitivity analyses, we further adjusted for potential confounders such as hypertension, total cholesterol, hypertriglyceridemia, leptin, and the number of *APOE* ε4 alleles. Leptin was log-transformed before analysis to reduce skewness. To further illustrate the nonlinear association with risk of dementia, we performed proportional hazard regression for insulin parameterized in terms of restricted cubic splines^30^ with 4 knots, automatically placed at 6.6, 10.4, 14.4, and 25.3 mIU/L. The median value of 12.3 mIU/L was chosen as the reference, and observations with the 1% lowest and 1% highest insulin values were excluded from the analysis. Post hoc analyses on quintiles of fasting insulin were performed to show the independence of associations on specific cut points (data available from Dryad, figure, doi.org/10.5061/dryad.21v1r72). To examine interactions between insulin and age, we also defined regression models with time from baseline to event or censoring as the underlying time scale and included age and age squared as predictor variables. The proportional hazards assumption was assessed with cumulative sums of martingale residuals (ASSESS option in the regression routine PHREG in SAS, SAS Institute Inc, Cary, NC), and variables were included only if there was no indication of a violation of the proportional hazard assumption (*p* > 0.05). Multicollinearity between predictors was assessed by separate linear models for survival until dementia diagnosis. Results presented here are based on regression models that did not show evidence for multicollinearity (variance inflation factors <2). All analyses were performed with SAS version 9.4 and MATLAB (R2016b; MathWorks, Inc, Natick, MA). Results with values of *p* < 0.05 were considered statistically significant (2-sided tests).

### Data availability

Anonymized data might be shared by request.

## Results

Baseline characteristics of participants are shown in table 1. Fasting insulin showed a larger variability than fasting glucose because the latter was restricted by an upper limit of 7.0 mmol/L in this sample of women without diabetes mellitus at baseline. Because of their association with diabetes mellitus, we also calculated estimates for insulin sensitivity and β-cell function to examine whether the state of low fasting insulin was a consequence of reduced production due to impaired β-cell function and preclinical diabetes mellitus. Insulin sensitivity and β-cell function were estimated from fasting values of insulin and glucose with HOMA, with insulin sensitivity being the inverse of HOMA insulin resistance.^25^ Because of restrictions regarding the values of fasting insulin and glucose, the number of observations for HOMA variables was reduced to 1,169. Mean BMI was in the range of normal weight, and the prevalence of obesity was 8%. Most of the participants had only basic education, which was the norm for women born during the first decades of the 20th century. More than 80% of women reported regular physical activity, and almost half of the sample reported ever smoking.

**Table 1:**
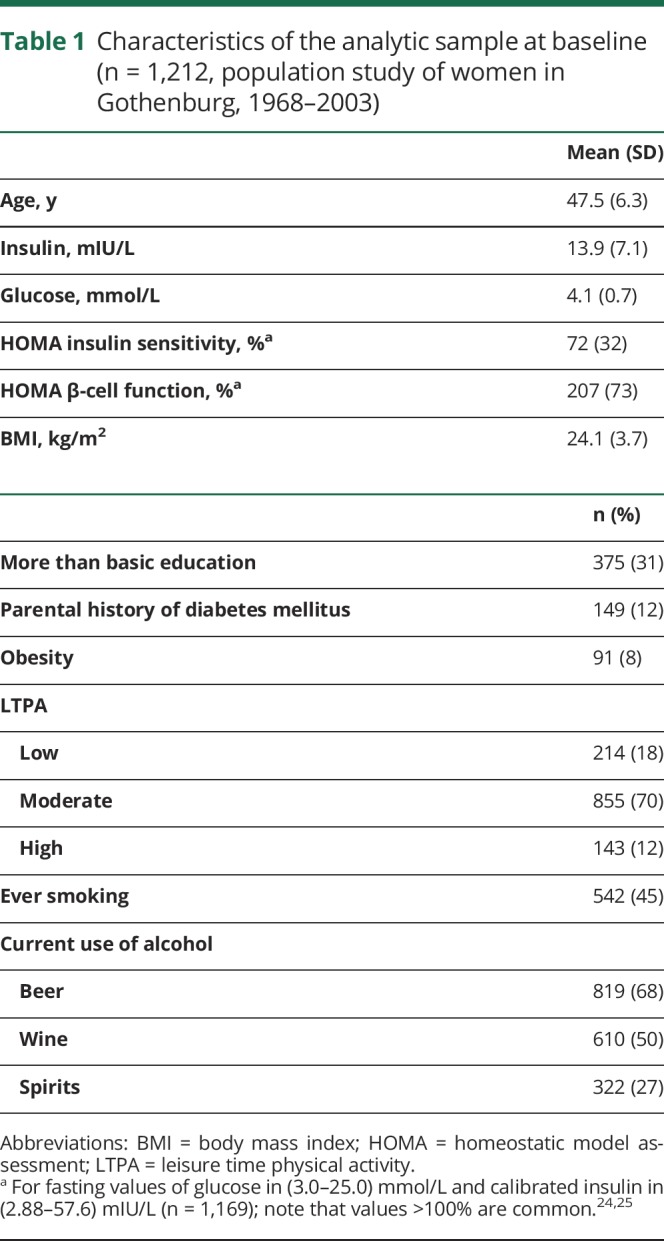
Characteristics of the analytic sample at baseline (n = 1,212, population study of women in Gothenburg, 1968–2003)

Table 2 shows the association between tertiles of insulin mutually adjusted for tertiles of glucose with 3 different endpoints, namely incident dementia, dementia censored for diabetes mellitus, and diabetes mellitus (panel A). Being in the low tertile of fasting insulin more than doubled the risk of dementia compared to being in the medium tertile, HR = 2.34 (1.52–3.58), while the high tertile was not significantly associated with higher risk of dementia. These associations were also seen with respect to dementia outcomes without a lifetime diagnosis of diabetes mellitus (column 2). In contrast, high but not low insulin was associated with a higher risk of diabetes (column 3). Compared to the medium tertile, the high tertile of glucose predicted dementia and was marginally associated with higher risk of diabetes mellitus. Panel B shows that lower values of HOMA insulin sensitivity predicted diabetes mellitus in a dose-dependent way, while HOMA β-cell function was not associated with diabetes mellitus. Regarding dementia endpoints, reduced risk was seen for low and medium tertiles of HOMA insulin sensitivity compared to the high tertile. Lower values for HOMA β-cell function were associated with a higher risk of dementia.

**Table 2:**
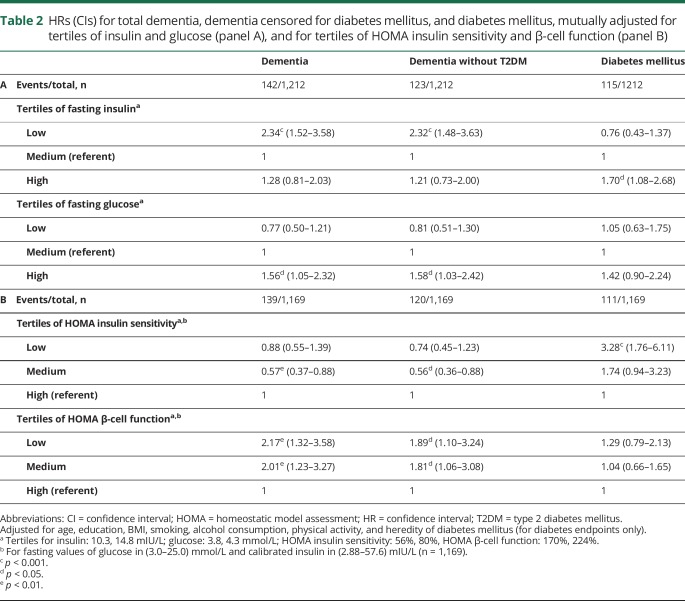
HRs (CIs) for total dementia, dementia censored for diabetes mellitus, and diabetes mellitus, mutually adjusted for tertiles of insulin and glucose (panel A), and for tertiles of HOMA insulin sensitivity and β-cell function (panel B)

The nonlinear association between fasting insulin and risk of dementia is further illustrated with the use of proportional hazard regression with restricted cubic splines (figure). We derived a value of 10.3 mIU/L for the approximate inflection point, below which the risk of dementia is increased. Post hoc analyses based on quintiles showed increased risk of dementia for women in the fifth quintile (fasting insulin ≥17.8 mIU/L) compared to the fourth quintile (HR 2.48; 95% confidence interval [CI] 1.31–4.71; data available from Dryad, figure, doi.org/10.5061/dryad.21v1r72).

**Figure:**
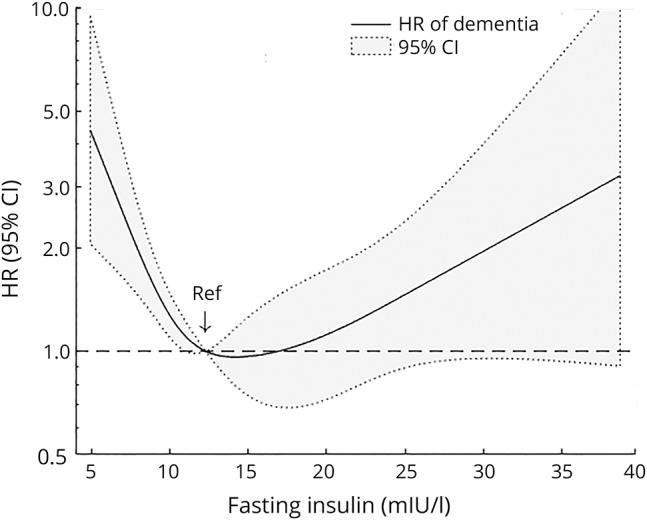
HR for dementia as a function of fasting insulin using restricted cubic splines Cubic spline regression model with 4 knots at 6.6, 10.4, 14.5, and 25.3 mIU/L, excluding the 1% lowest and 1% highest values of fasting insulin. Reference is set at 12.3 mIU/L (median). The model is adjusted for age, education, body mass index, smoking, alcohol consumption, physical activity, and tertiles of fasting glucose. The *p* value for test of nonlinearity is 0.0002. Artwork was performed with MATLAB (R2016b; MathWorks, Inc, Natick, MA). CI = confidence interval; HR = hazard ratio.

To investigate the association of fasting insulin and glucose with dementia subtypes while accounting for the smaller number of events, proportional hazard regression on tertiles of insulin and glucose was performed with stepwise selection of covariates from variables describing education, lifestyle, and BMI. For VD, low insulin predicted dementia independently of diabetes comorbidity (table 3). Additional predictors of VD were BMI and ever smoking, but BMI was no longer a predictor of VD censored for diabetes mellitus. AD was predicted by low insulin, and this association was strengthened after censoring for diabetes comorbidity. Other covariates did not predict AD. When participants with <20 years of follow-up were excluded in sensitivity analyses, the associations between variables describing insulin and glucose metabolism and dementia outcomes were strengthened (data available from Dryad, table 1, doi.org/10.5061/dryad.21v1r72).

**Table 3:**
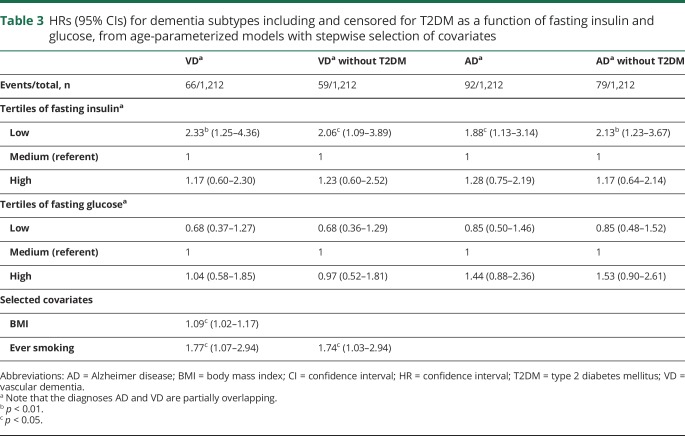
HRs (95% CIs) for dementia subtypes including and censored for T2DM as a function of fasting insulin and glucose, from age-parameterized models with stepwise selection of covariates

Table 4 gives the characteristics of covariates and the number of incident cases of diabetes mellitus, dementia, and mortality by fasting insulin tertile. Compared to women in the medium tertile of insulin, those in the low insulin tertile had lower values of glucose and HOMA β-cell function and higher values of HOMA insulin sensitivity. They also had lower BMI, blood pressure, and heart rate; had lower values of triglycerides and leptin; and were less likely to be hypertensive. They were more likely to have higher education, to be physically active, to be a smoker, or to consume alcohol. *APOE* ε4 allele frequency was higher in the low insulin tertile compared to higher tertiles. There was no difference in diabetes incidence but a higher incidence of dementia in the low compared to the medium tertile. In contrast, being in the high insulin tertile was associated with high values of cardiovascular risk factors and with excess risk of diabetes mellitus. Total mortality showed a U-shaped risk curve with respect to insulin. A corresponding analysis of tertiles of fasting glucose showed fewer contrasts with regard to covariates and endpoints (data available from Dryad, table 2, doi.org/10.5061/dryad.21v1r72). The age-adjusted analysis demonstrated excess risk for diabetes mellitus in the high glucose tertile, although this is highly dependent on insulin and BMI, as shown in the mutually adjusted model (table 2; data available from Dryad, table 3).

**Table 4:**
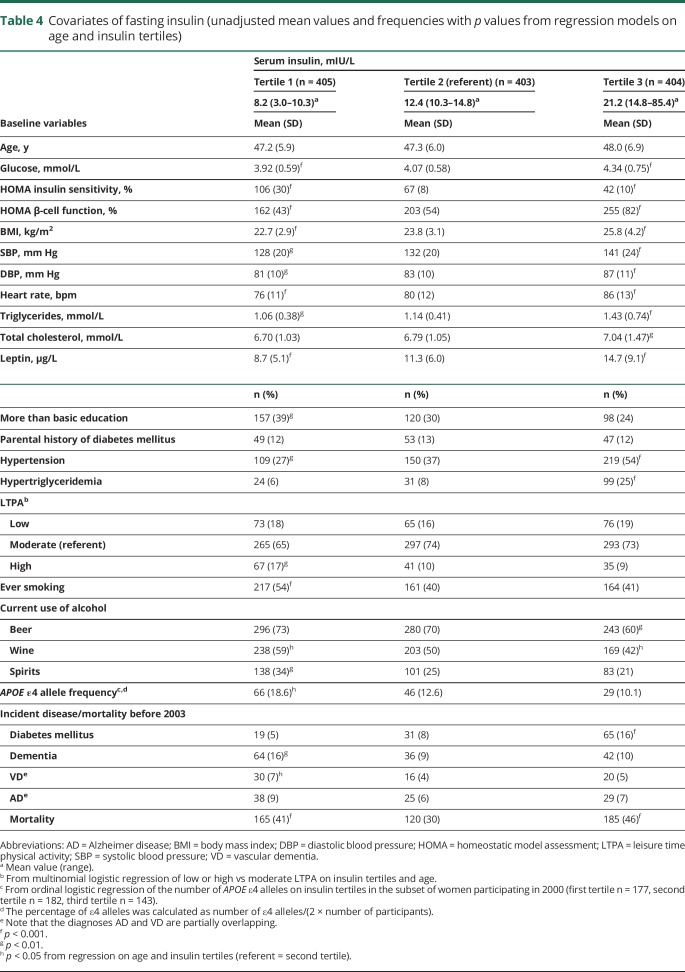
Covariates of fasting insulin (unadjusted mean values and frequencies with *p* values from regression models on age and insulin tertiles)

Further adjustment of the model for dementia for variables associated with insulin hardly changed the estimates for insulin and glucose given in table 2. Among these potential confounders, only hypertriglyceridemia (HR 1.96, 95% CI 1.23–3.12) and higher cholesterol (HR 1.14, 95% CI 1.04–1.25) predicted dementia (data available from Dryad, table 4, doi.org/10.5061/dryad.21v1r72). Adjusting for *APOE* genotype restricted the sample to those participants who survived until 2000 (n = 502). To reduce survival bias, we excluded the oldest women (60 years old at baseline, n = 4) ensuring that there was no age difference by insulin tertile. In this subsample, low insulin predicted dementia (HR 3.01, 95% CI 1.56–5.84) compared to the medium insulin tertile independently of higher *APOE* ε4 allele frequency (HR 2.18, 95% CI 1.29–3.67) per allele (data available from Dryad, table 4, right column).

## Discussion

In this population-based study of middle-aged women free of diabetes mellitus at baseline, the 34-year risk of dementia was more than doubled in women with initially low fasting serum insulin compared to women with medium insulin values. The association was independent of fasting glucose, BMI, education, and lifestyle factors and was strengthened when follow-up was restricted to ≥20 years, refuting the hypothesis that low insulin was a consequence of weight loss often seen in the prodromal phase of dementia. The association with low fasting insulin was also seen when dementia without diabetes comorbidity was the outcome of interest, considering dementia with diabetes mellitus as a competing event. Regarding incident diabetes mellitus, the largest risk was observed for high vs medium values of fasting insulin, and no risk at all was seen for low insulin. Low insulin values at the baseline of the 34-year follow-up thus cannot be interpreted as a sign of reduced insulin secretion due to preclinical T2DM. This conclusion is supported by the observation that low HOMA insulin sensitivity predicted incident diabetes mellitus, but low HOMA β-cell function did not. These findings suggest that the state of low fasting insulin characterizes an alternative pathway to dementia that is different from the one via hyperinsulinemia and T2DM.

While risk of dementia due to hyperinsulinemia has been shown previously,^1,3^ the present study is the first to replicate the observation of an adverse association between low fasting insulin values and dementia, which was described in a cohort of elderly men followed up over 5 years.^5^ Our results generalize this finding to women while enhancing the evidence for a causal association because the 34-year follow-up, with a minimum of 20 years in sensitivity analyses, decreased the probability of reverse causation for dementia. Third, the observation that low insulin predicted dementia without diabetes comorbidity, and not diabetes mellitus, makes it possible to identify hypoinsulinemia as a risk factor for dementia that is fundamentally different from hyperinsulinemia or diabetes mellitus. This was in contrast to the previously mentioned study in which about one-third of all men had diabetes mellitus at baseline and incident diabetes mellitus was not accounted for.^5^ In addition, the women in the present study were recruited in the late 1960s when the prevalence of obesity was still low. It is possible that the proportion of subjects with low insulin was relatively large compared to modern cohorts, which may have made it more likely to observe the association with dementia in the present sample.

The phenotype associated with low insulin was characterized by low body weight, low blood pressure and heart rate, and low values of glucose, triglycerides, and leptin. These associations were not explained by cigarette smoking or high physical activity, lifestyle variables that were also associated with low insulin in both this study and previous studies.^31,32^ Twenty years ago, Bonora et al.^33^ described a U-shaped risk curve for fasting insulin and coronary heart disease in a cross-sectional study. They found similar correlates with low insulin as demonstrated here and postulated that this condition, despite high insulin sensitivity, presented a state of insufficient cell insulinization, promoting the development of atherosclerosis. If low serum insulin is a marker for low insulin in the brain, it may, through its role as a mitogenic growth factor, cause impaired neuronal functioning, cell death, and dementia.^3,7^ This interpretation is supported by recent in vivo clinical results showing that low levels of fasting blood insulin were associated with increased β-amyloid deposition and neurodegeneration in nondiabetic, cognitively normal older adults.^34^ Consistent with these findings, the association between low insulin and dementia described here was particularly strong for VD and for AD without diabetes comorbidity, i.e., when the alternative pathway through diabetes mellitus was censored. Although insulin concentrations in the CSF are generally well below serum levels, the ratio varies between 1% and 10%, depending on weight status and other factors that regulate the transport across the blood-brain barrier^8^ and influence brain insulin levels in addition to absolute levels of circulating insulin. However, sensitivity analyses showed that none of the covariates that correlated with serum insulin explained the reported associations with dementia endpoints, underlining the fundamental importance of insulin for the etiology of dementia.

Compared with the risk due to low fasting insulin, the excess risk due to high insulin was less pronounced in this study, which may be due to the secular changes in BMI as mentioned above and the fact that women with T2DM at baseline were excluded from the sample. Compared to the other 2 insulin tertiles, the high insulin tertile had a low prevalence of the *APOE* ε4 allele and a high incidence of diabetes mellitus, which is consistent with observations that diabetes mellitus as a risk factor for dementia is more often observed in noncarriers of the *APOE* ε4 allele.^4,35,36^

The main strengths of this study are the assessment of risk factors in midlife, the long follow-up that reduces the risk of reverse causation due to preexisting dementia, and the high quality of endpoint data regarding both diabetes mellitus and dementia. A limitation of the study is that insulin was measured in serum samples that had been stored at −20°C for 45 years. Although validated in a subsample with baseline measurements in fresh samples, values were generally underestimated.^23^ Nonparametric regression models were used to reduce the influence of misclassification, but precise cut points for the U-shaped association with dementia should be derived from studies using fasting insulin obtained in fresh samples. In view of the pulsatile mode of insulin secretion even in the fasting state,^37^ the lack of more than 1 insulin measurement is acknowledged as further limitation. Moreover, the small number of endpoints available in the 34 years of follow-up limits our power to study subtypes of dementia (AD, VD, dementia with and without diabetes comorbidity) and interactions between, for example, insulin and glucose tertiles. Finally, we note that the *APOE* genotype was known only in participants who survived until 2000, which may have biased the associations between fasting insulin, genotype, and dementia.

This study provides epidemiologic evidence for a new pathway to dementia that is characterized by low fasting serum insulin and differs from the metabolic pathway via hyperinsulinemia or diabetes mellitus. The identification of the tails of the U-shaped risk curve for insulin and dementia with distinct mechanisms was possible because of the long follow-up and by comparing the associations of insulin with different dementia endpoints and diabetes mellitus. The distinction between alternative pathways may explain inconsistent epidemiologic findings regarding risk factors of dementia and its subtypes, as well as open new avenues for prevention.

## Glossary

AD: Alzheimer disease
BMI: body mass index
CI: confidence interval
DSM-III-R: *Diagnostic and Statistical Manual of Mental Disorders, 3rd edition, revised*
HOMA: homeostatic model assessment
HR: hazard ratio
LTPA: leisure time physical activity
T2DM: type 2 diabetes mellitus
VD: vascular dementia
